# Draft genome sequence of *Methylobacterium* sp. OT2 isolated from human skin and capable of growing on *t*-octylphenol polyethoxylate

**DOI:** 10.1128/mra.01193-23

**Published:** 2024-02-22

**Authors:** Haelim Son, Shir-Ly Huang, Kyoung Lee

**Affiliations:** 1 Department of Bio Health Science, Changwon National University, Changwon, South Korea; 2 Institute of Microbiology and Immunology, National Yang Ming Chiao Tung University, Taipei, Taiwan; Loyola University Chicago, Chicago, USA

**Keywords:** *Methylobacterium*, OT2, human skin, genome analysis

## Abstract

Here, we present the draft whole-genome sequence of *Methylobacterium* sp. OT2, isolated from human skin on a minimal medium containing *t*-octylphenol ethoxylate (Triton X-100). This genomic information contributes to understanding the niche adaptation on human skin and its catabolism of Triton X-100 in this strain.

## ANNOUNCEMENT

*Methylobacterium* strains are common on human skin, but their genomic information is limited (1, 2). Here, we report the genome sequence of a *Methylobacterium* strain isolated from human skin. A forehead skin swab from a female college student was streaked on minimal salts basal medium agar (3) with 0.5% Triton X-100 as a carbon and energy source. After 1 week of aerobic incubation at 28°C, one isolate, OT2, was purified on Nutrient agar (KisanBio Co) and incubated for 4 days at 28°C. The strain was deposited in the Korean Collection for Type Cultures as KCTC 82560 and Japan Collection of Microorganisms as JCM 34944 (NCBI taxonomy ID 2813779). Ethical approval for subject sampling was granted by the institutional review board of Changwon National University. Sanger sequencing of the 16S rRNA gene using primers 27F (5′-AGAGTTTGATCCTGGCTCAG-3′) and 1492R (5′-GGTTACCTTGTTACGACTT-3′), along with BLASTN analysis (4), identified OT2 as a member of the genus *Methylobacterium*. It showed the highest similarity to *M. oryzae* CBMB20^T^ (99.63%) and *M. fujisawaense* strain DSM 5686^T^ (99.63%).

For DNA extraction, cells were cultured in a flask on Nutrient broth with 0.1% sodium pyruvate for 48 h at 28°C with shaking at 140 rpm. Bacterial cells were washed and boiled for 1 min, followed by lysozyme and mutanolysin for 1 h at 37°C and then proteinase K, SDS, and RNase I for 1 h at 56°C. The total DNA from the mixture was purified using a conventional method that involves phenol/chloroform extraction and salt/ethanol precipitation, followed by dissolution in Tris-EDTA buffer (pH 8.0) (5). Genomic DNA was sequenced using Illumina and Oxford Nanopore Technologies MinION platforms. Illumina sequencing at DNALink Co. (Seoul, Korea) employed a TruSeq DNA PCR-free 550-bp library kit (Illumina), and demultiplexing was performed by bcl2fastaq2 (ver. 2.20) on the Illumina NovaSeq6000 sequencer. Raw sequencing data quality was assessed using FastQC with ASCII Qscore offset 33 (https://www.bioinformatics.babraham.ac.uk/projects/fastqc/) (6), showing a 2 × 151 read length with an approximately 550-bp insert size and a mean Phred quality score of 35.25. For Nanopore sequencing, libraries were prepared with the SQK-RBK004 kit, multiplexed using the EXP-NBD104 barcoding kit, and sequenced on a MinION sequencer (v20.10.3) with a FLO-MIN106 Flow Cell Type, using default settings on a Precision-7920-Tower workstation. Reads were base-called and demultiplexed using Guppy v4.2.2 in fast accuracy mode, with min_qscore = 7 as read filtering. The reads underwent *de novo* hybrid assembly using Unicycler v0.4.9b, incorporating the SPAdes v. 3.12.0 program with default settings (7). Gene predictions and annotations were conducted by NCBI, utilizing the best-placed reference protein set, GeneMarkS-2+ of the NCBI Prokaryotic Genome Annotation Pipeline 6.5 (8).

The final assembly yielded a genome size of 6,564,979 bp distributed across eight contigs, with three forming a circular structure. Table 1 outlines general genome sequencing and assembly features. The NCBI assembly report (ASM2357356v1) showed that OT2 exhibited the highest similarity to *M. radiotolerans* NBRC 15690^T^ (NCBI RefSeq assembly GCF_007991055), with an average nucleotide identity (ANI) value of 92.7% and 81.65% assembly coverage. Considering the 95%–96% ANI threshold for bacterial species delineation (9), OT2 may represent a new species within the *Methylobacterium* genus.

**TABLE 1:** General features of the *Methylobacterium* sp. OT2 genome sequencing and assembly

Feature		
Isolation		Human skin
Sequencing analysis		
Illumina	No. of reads	33,689,170
	Total read length (Mbp)	5,087
Nanopore	No. of reads	3,144
	Total read length (Mbp)	18.5
	Mean read length (standard deviation) (bp)	5,877 (14,764)
	N_50_ (bp)	25,352
Assembly analysis		
	No. of contigs (no. circular contigs)	8 (3)^*a*^
	Size (bp)	6,564,979
	GC content (%)	71.1
	Genome coverage (×)	777.7
	No. of protein coding genes	5,998
	No. of rRNA genes (5S, 16S, and 23S)	5
	No. of tRNA genes	57
	No. of ncRNA genes	5
	Estimated completeness (%)	100.0
	Estimated contamination (%)	0.25

^*a*^ The three circular contigs, namely, pOT2-1, pOT2-2, and pOT2-3, have sizes of 550,364 bp, 44,788 bp, and 13,421 bp, respectively. pOT2-1 harbors the *parABC* genes involved in plasmid partitioning. pOT2-2 carries a gene for a plasmid partitioning protein of the ParA family and a gene for a recombinase family protein. pOT2-3 contains a gene for a partitioning protein of the ParA family and two genes for recombinase family proteins.

## Data Availability

The Whole Genome Shotgun project has been deposited in GenBank under accession no. NZ_JAFIWG000000000. The SRA accession numbers used for assembly are SRX10146467 (Illumina Nova seq) and SRX10146468 (MinIon seq).
